# Attenuation of Post-Exercise Energy Intake Following 12 Weeks of Sprint Interval Training in Men and Women with Overweight

**DOI:** 10.3390/nu14071362

**Published:** 2022-03-24

**Authors:** Natalya J Beer, Ben Jackson, James A Dimmock, Kym J Guelfi

**Affiliations:** 1 School of Human Sciences (Exercise & Sport Science), The University of Western Australia, Perth 6009, Australia; ben.jackson@telethonkids.org.au (B.J.); kym.guelfi@uwa.edu.au (K.J.G.); 2 Telethon Kids Institute, Perth 6009, Australia; 3 Department of Psychology, College of Healthcare Sciences, James Cook University, Townsville 4810, Australia; james.dimmock@jcu.edu.au

**Keywords:** appetite, food consumption, ghrelin, high-intensity interval exercise, energy balance

## Abstract

An acute bout of sprint interval training (SIT) performed with psychological need-support incorporating autonomy, competence, and relatedness has been shown to attenuate energy intake at the post-exercise meal, but the long-term effects are not known. The aim of this trial was to investigate the effects of 12 weeks of SIT combined with need-support on post-exercise food consumption. Thirty-six physically inactive participants with overweight and obesity (BMI: 29.6 ± 3.8 kg·m^−2^; $\dot{V}O_{2\mathrm{peak}}$ 20.8 ± 4.1 mL·kg^−1^·min^−1^) completed three sessions per week of SIT (alternating cycling for 15 s at 170% $\dot{V}O_{2\mathrm{peak}}$ and 60 s at 32% $\dot{V}O_{2\mathrm{peak}}$) with need-support or traditional moderate-intensity continuous training (MICT) without need-support (continuous cycling at 60% $\dot{V}O_{2\mathrm{peak}}$). Assessments of appetite, appetite-related hormones, and ad libitum energy intake in response to acute exercise were conducted pre- and post-intervention. Fasting appetite and blood concentrations of active ghrelin, leptin, and insulin did not significantly differ between groups or following the training. Post-exercise energy intake from snacks decreased significantly from pre- (807 ± 550 kJ) to post- SIT (422 ± 468 kJ; *p* < 0.05) but remained unaltered following MICT. SIT with psychological need-support appears well-tolerated in a physically inactive population with overweight and offers an alternative to traditional exercise prescription where dietary intake is of concern.

## 1. Introduction

Regular exercise is important for overall health and well-being and is widely recommended for weight loss and maintenance. Specifically, regular exercise may assist with creating a negative energy balance through increasing energy expenditure and/or improving the sensitivity of appetite regulation [1]; however, these responses may vary across individuals and conditions [2]. In particular, the relationship between acute exercise and subsequent food/drink consumption appears to be influenced by both the format of, and psychological experiences in, exercise [3,4]. Whether this translates to differences in appetite and energy intake in the long term remains to be determined.

The effect of exercise format on appetite responses has been examined in a number of studies with comparisons often made between high-intensity intermittent exercise (HIIT; incorporating target intensities between 80% and 100% peak heart rate or peak oxygen consumption ($\dot{V}O_{2\mathrm{peak}}$)) or sprint interval exercise (SIT; differentiated from HIIT by workloads prescribed at a supramaximal level) [5] and traditional moderate-intensity continuous exercise (MICT). Acutely, single bouts of HIIT or SIT have been associated with both lower overall post-exercise energy intake [6] and lower consumption of ‘unhealthy’ foods [4] compared with moderate-intensity continuous training (MICT), of matched duration (30 min) and total work. Beyond acute exercise, Sim and colleagues [7] observed a tendency for more sensitive appetite regulation (i.e., a greater difference in food intake in response to a high- or low-energy preload meal) following 12 weeks of SIT but not traditionally recommended MICT, in men with overweight. Alkahtani and colleagues [8] and Panissa and colleagues [9] noted a decrease in exercise-induced hunger and desire to eat, together with a decrease in fat intake, following four and six weeks, respectively, of HIIT compared with MICT. However, others have observed similar perceptions of fasting appetite [10], and three-day self-reported energy intake [11] following programs of HIIT compared with MICT, and a recent meta-analysis of the effect of interval training on energy intake revealed no significant differences in energy intake following varying interventions of HIIT or SIT and MICT [12]. Importantly, all but one of the 16 studies included in this analysis relied on self-report measures of food intake, such as food diaries or food frequency questionnaires, which may provide erroneous and/or biased results [13], particularly given that participants in many of the included studies were instructed to maintain their habitual food consumption. The heterogeneity of energy intake assessment, together with the varied interval training protocols studied, suggests that conclusions about the efficacy of interval training protocols to influence appetite and food choices may be premature.

With respect to psychological experiences in exercise, researchers have shown that factors associated with different forms of exercise motivation may influence subsequent food consumption. From the lens of the Self-Determination Theory (SDT) [14], individuals may undertake exercise because they fully endorse the instrumental outcomes of the activity (i.e., “identified regulation”), because it is aligned with their values and identity (i.e., “integrated regulation”), and/or because they enjoy the process of exercising (i.e., “intrinsic motivation”). When primarily citing these reasons for engaging in exercise, individuals are considered to possess autonomous motivation. For some individuals, however, exercise is undertaken to obtain externally imposed rewards or to avoid externally imposed punishments (i.e., “external regulation”), and/or due to internal pressures such as to avoid feelings of guilt or anxiety (i.e., “introjected regulation”). When primarily governed by external regulation and/or introjected regulation, individuals are considered to possess controlled motivation. Conceptual and empirical work indicates that controlled (as opposed to autonomous) motivation for exercise may be associated with cognitive and physiological factors that increase the desire for hedonically pleasurable and ‘unhealthy’ foods/drinks [15,16]. Additionally, it has been suggested that high-quality motivation for exercise may have a ‘spill-over’ effect on other health behaviors, such as dietary intake [17]. Indeed, it is possible to promote individuals’ autonomous motivation for exercise by providing social conditions that are supportive of three psychological needs [18]. These needs are for autonomy (i.e., the need to feel that one’s behavior is self-regulated), relatedness (i.e., the need to feel that one is meaningfully connected with others), and competence (i.e., the need to experience a sense of accomplishment when striving for personally relevant and challenging goals). Of relevance, providing a key component of autonomy-support—that of choice—during an acute bout of exercise has been shown to attenuate total and unhealthy energy intake following exercise [19]. Research is needed, however, to investigate the full effects of need-supportive exercise environments on post-exercise eating behaviors.

It is evident that further investigations are required to better understand the effect of both exercise format (specifically SIT) and psychological experiences during exercise on subsequent energy intake, but little is also known about the potential interaction of these factors beyond the acute setting. Such an interaction was recently observed whereby total energy intake at a post-exercise test meal was lower following SIT compared with MICT only when exercise was delivered with support for individuals’ psychological needs of autonomy, competence, and relatedness [4]. Such an interaction of the format of exercise and psychological experiences may explain, at least in part, the heterogeneity of eating behaviors around exercise, with some exercise protocols attenuating subsequent food consumption; but only when administered in such a way that optimizes individuals’ psychological experiences (e.g., through social conditions that influence psychological experiences). Questions remain, however, about the efficacy of prolonged participation in these exercise conditions, which combine manipulations to both the format and psychological experiences of exercise, to influence energy intake and food choices. As such, the primary objective of this trial was to investigate the effects of a 12-week exercise intervention incorporating SIT, with the addition of need-support, compared with standard exercise guidelines (i.e., MICT without need-support) on post-exercise food consumption. Secondary outcomes included fasting and post-exercise appetite, and appetite-regulating blood variables. The response of fitness, body composition, and self-reported eating behavior to the training intervention was also compared. It was hypothesized that 12 weeks of SIT would result in lower post-exercise food consumption relative to MICT and a fasting hormonal milieu associated with attenuated energy intake.

## 2. Materials and Methods

### 2.1. Participants

**Figure 1:**
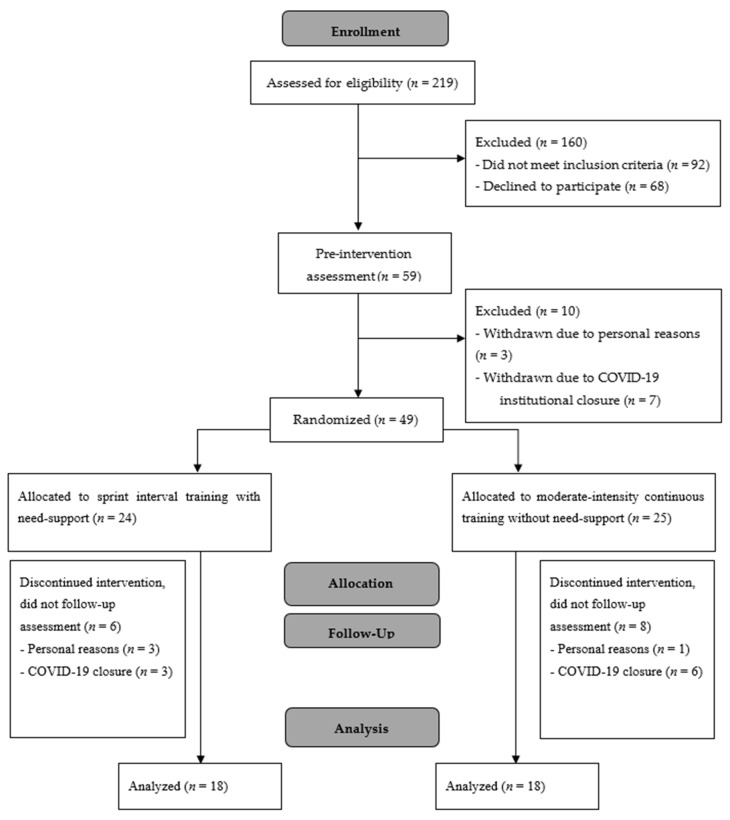
CONSORT flow diagram.

Individuals were eligible for participation if they were aged between 18 and 40 years, physically inactive (defined as performing ≤ 75 min of moderate to vigorous physical activity per week), and had a BMI of ≥25 kg·m^−2^. Exclusion criteria were a history of medical conditions such as diabetes, cardiovascular disease, and/or eating disorders known to affect appetite and food intake, a score of ≥3.5 on the restraint scale of the Dutch Eating Behavior Questionnaire [20], injury or illness limiting the ability to exercise, current medication which would interfere with appetite, dietary restriction (e.g., vegan, currently dieting to lose weight, etc.), and pregnancy or planned pregnancy during the study. Fifty-nine eligible individuals were recruited into the study and completed the initial assessments. Of these, 16 participants were unable to continue in the study due to the timing of institutional closures associated with COVID-19, and seven discontinued for personal reasons (*n* = 6 SIT; *n* = 7 MICT; *n* = 10 not yet randomized), leaving 36 participants (men = 6; women = 30) who completed the study (Figure 1). A total sample size of 28 was expected to provide sufficient power (95%) to detect significant differences in the primary outcome of post-exercise energy intake (*p* < 0.05) based on previous work [4] in which a large effect size (d = 0.73) was observed for differences in energy intake following an acute bout of SIT with need-support and MICT. The study was approved by the Institutional Human Ethics Committee, and written consent was obtained from all participants; however, to minimize the potential for biased responses, participants were not informed that their food intake was being assessed in the study. Instead, they were informed that the aim of the study was to investigate the effect of regular exercise training on well-being and markers of stress in the body. Participants were probed for suspicion by answering the single item “In your own words, please describe the purpose of this study”, which confirmed that no participants suspected the assessment of energy intake. All participants were debriefed as to the true purpose of the study upon completion of data collection.

### 2.2. Study Design

Using a between-subjects yoked design (see, e.g., [21]), each participant was required to attend two testing sessions pre- and post-training and three training sessions per week for 12 weeks. The first session was completed within two weeks of commencing the training and included baseline assessments of motivational orientations towards exercise, current exercise behaviors, eating habits and food preferences, fitness, and anthropometry (see “Visit 1” section for further details). Following this session, participants were pair-matched based on sex, age (±5 years), $\dot{V}O_{2\mathrm{peak}}$ (±5 mL·kg·min^−1^), body mass (±5 kg), height (±10 cm), and fat mass (±5 kg). Within each pair, one participant was randomly allocated, using a random number generator, to one of two conditions: SIT with need-support or MICT without need-support, with the other participant yoked to the alternative condition. These conditions were selected were based on the difference seen in post-exercise energy intake following an acute bout of exercise in our previous work [4]. The second visit, which was completed within one week of commencing the training, involved the assessment of appetite and energy intake in response to a 30-min bout of exercise performed at the same relative intensity and format of their prescribed training sessions (see “Visit 2” section for further details). This method was chosen (as opposed to other assessments of energy intake, such as the pre-load test paradigm) in order to assess responses to both physiological and psychological manipulations to an acute bout of exercise pre- and post-intervention.

### 2.3. Exercise Training

Participants were required to complete three supervised training sessions per week over 12 weeks. All training was conducted on front-access air-braked cycle ergometers (Model EX-10, Repco Cycle, Huntingdale, Victoria, Australia) interfaced with a customized software program (Cyclemax, School of Human Sciences, University of Western Australia, WA, Australia). The exercise comprised of either: (i) SIT, alternating high- and low-intensity efforts performed at a ratio of 1:4 (15 s at a power output equivalent to 170% $\dot{V}O_{2\mathrm{peak}}$) with an active recovery period (60 s at a power output of 32% $\dot{V}O_{2\mathrm{peak}}$) between efforts, with the addition of psychological need-support or (ii) MICT, continuous cycling at 60% $\dot{V}O_{2\mathrm{peak}}$, performed without psychological need-support (i.e., standard exercise recommendations). The workload for each participant was determined using their individual baseline $\dot{V}O_{2\mathrm{peak}}$ results. Need-support was provided to participants randomized to the support group through a number of techniques outlined in an expert consensus study [22] and described previously [4]. For example, autonomy was supported by providing clear rationales and benefits of the exercise, offering choices where possible (e.g., of the music accompaniment), inviting questions, and using non-controlling language. Competence was supported by offering positive, relevant feedback and encouraging goal-setting. Relatedness was supported by offering empathy where appropriate and displaying appreciation and concern for participants’ well-being. Autonomy, competence, and relatedness were not intentionally supported in the no-support condition; however, to increase the ecological validity of the study, no attempts were made to purposely undermine participants’ experiences (i.e., participants in the no-support condition received ‘neutral’ exercise conditions). These conditions were chosen to compare the training effects of the SIT protocol that resulted in greater suppression of food intake seen in our previous research [4] with a ‘standard’ exercise situation.

All sessions were performed individually and supervised by a trained exercise instructor. Training sessions commenced with a 3-min warm-up, which involved cycling at 50 W. To accommodate for any increase in fitness throughout the exercise training, the duration of training sessions was progressively increased as follows: weeks 1–2 30 min (i.e., 24 sets of work:rest in SIT) weeks 3–4 35 min (i.e., 28 sets of work:rest in SIT), weeks 5–6 40 min (i.e., 32 sets of work:rest in SIT), weeks 7–8 45 min (i.e., 36 sets of work:rest in SIT), weeks 9–12 50 min (i.e., 40 sets of work:rest in SIT). Training workloads were adjusted following a $\dot{V}O_{2\mathrm{peak}}$ test performed during week 6 of the exercise training. The prescribed exercise intensities were confirmed by monitoring cycling power output and total work during each session. Ratings of perceived exertion (RPE) [23] and HR were collected every 10 min during exercise, while perceptions of need-support were assessed via a 15-item questionnaire administered upon completing the training intervention [18]. This instrument contained items assessing autonomy-, structure/competence-, and involvement/relatedness-support. Responses were scored on a five-point scale ranging from 0 (not true for me) to 4 (very true for me). The Intrinsic Motivation Inventory (IMI) [24] was also administered to assess participants’ perceived enjoyment, choice, competence, and relatedness during the exercise training using a seven-point response scale anchored at 1 (not true at all) to 7 (very true).

### 2.4. Outcome Measures

Outcome measures were assessed during two separate testing sessions conducted both pre- and post-training. The first visit included assessments of behavioral characteristics, aerobic fitness, and body mass and composition, and was completed between 24 h and 1 week following cessation of the training. In the second visit, participants completed 30 min of standardized exercise, and the subsequent responses of appetite, energy intake, and free-living physical activity were monitored. The primary outcome of the study was post-exercise energy intake, with all other outcomes considered secondary.

Visit 1: Assessment of fitness, body composition, and behavioral characteristics

Approximately two weeks prior to commencing training, each participant attended the laboratory for assessment of motivational orientation toward exercise (Behavioural Regulation in Exercise Questionnaire-3) [25], compensatory eating (Compensatory Eating Motives Questionnaire) [26], and post-exercise unhealthy snack licensing (Exercise Snacking Licensing Scale) [16]. Peak oxygen consumption was measured using a continuous graded exercise test on an air-braked front-access cycle, as described previously [4]. Body composition (specifically fat mass and lean mass) was assessed via dual-energy X-ray absorptiometry (Lunar iDXA, GE Healthcare, Madison, WI, USA). All assessments were repeated within one week of training completion.

Visit 2: Assessment of appetite, energy intake, and free-living physical activity

**Figure 2:**
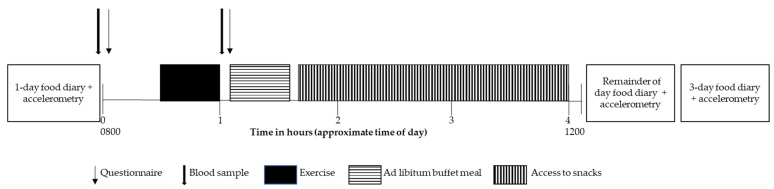
Visit 2 timeline.

Approximately one week prior to and following the exercise training, participants attended the laboratory at 0800 h, after an overnight fast, having consumed 300 mL of water upon waking (Figure 2). Women were tested in the follicular phase of the menstrual cycle (day 7 ± 3, as determined by the onset of menstruation) given the effect of the menstrual cycle on appetite and energy intake [27]. Free-living food consumption was determined via self-report food diaries completed on the day before visit 2 (to ensure prior dietary consistency) and for the subsequent 3 days (four days total to determine the mean free-living intake). Instructions on the use (including a one-day example) and the necessity for accurate and detailed recordings of food and/or drink intake immediately after consumption were emphasized. The total kilojoules ingested were calculated using a commercially available software program (FoodWorks v 4.2.0, Xyris Software, Qld, Australia). Baseline assessments were taken (outlined below), after which participants completed 30 min of either MICT without need-support or SIT with need-support (as per their allocated training intervention). 

Perceived appetite was assessed pre- and post-exercise using a modified visual analogue scale. This validated scale took the form of two straight lines (100 mm in length each) accompanied by a question anchored with words representing extreme states of hunger or fullness [28]. At the same timepoints, capillary blood was sampled to determine fasting and post-exercise concentrations of appetite-related blood variables, including glucose, lactate, active ghrelin, leptin, and insulin using methods described previously [4]. The hormones measured were selected based on their role in the episodic (ghrelin) and tonic (insulin and leptin) regulation of appetite [29]. The intra-assay coefficient of variation was 10.9% for ghrelin, 13.7% for leptin, and 10.3% for insulin.

Twenty min post-exercise, participants were provided with access to a laboratory test meal. The initial laboratory test meal consisted of products of known and differing macronutrient composition, including an assortment of typical breakfast foods and treats such as bread, spreads, cereal, milk, fruit, muffins, and biscuits, which was available for 30 min. Following this, and for the remaining 2.5 h of monitoring, participants remained resting in the had free access to a number of typical snack items (e.g., fruit, chocolate, salted chips/crisps). Water was not offered to participants during the exercise; however, a standardized bottle of plain drinking water (~1500 mL) was made available during this monitoring period afterwards. To determine energy intake, the post-consumption weight was subtracted from the pre-meal weight of each food item. The amount of food consumed (grams) was multiplied by the number of kilojoules within the product, as specified by the manufacturer’s nutrition label, or by the FoodWorks software package where nutrition labels were not available. In order to classify foods as ‘healthy’ and ‘unhealthy’, participants rated all the food provided on a scale anchored at 1 (very unhealthy) to 7 (very healthy). Foods that scored on average below the midpoint of the scale (i.e., 3.5) were classified as ‘unhealthy’ (confectionary, muffins, chocolate biscuits, and Coco Pops breakfast cereal; 1.8 ± 0.2) and vice versa for ‘healthy’ foods (fruits, low-fat milk, Sanitarium Weetbix breakfast cereal, wholemeal bread, and low-fat spreads; 4.6 ± 0.5). These participant-derived classifications were verified by ratings obtained from an independent dietitian who was blind to the study purpose (i.e., the same foods were classified as healthy and unhealthy).

Free-living physical activity was assessed using an accelerometer (GT3X+ Activity Monitor, ActiGraph, Pensacola, FL, USA) worn on the right hip for four days starting the day of visit 2 pre- and post-training. Valid wear time was considered 10 h per day, and data were recorded in 60 s epochs. Energy expenditure was determined using the ActiLife software (version 6.9.3, 2014, Pensacola, FL, USA). 

### 2.5. Statistical Analyses

Analyses were conducted using the SPSS version 25 software package for Windows, with statistical significance being accepted at an alpha level of *p* < 0.05. To assess whether the background characteristics of participants randomized to the two groups differed prior to the exercise training, univariate analysis of variance (ANOVA) was conducted to compare age, body mass, height, BMI, $\dot{V}O_{2\mathrm{peak}}$, fat mass, lean mass, and baseline activity levels (i.e., mean daily energy expenditure). One-way repeated-measures ANOVAs were applied to determine differences in mean HR, RPE, and psychological perceptions of the exercise training. Two-way (condition × time) ANOVAs were applied to determine the effects of the exercise training on fitness, body composition, and free-living energy expenditure (as measured by accelerometry). The effect of the exercise training on exercise motivation, compensatory eating, snacking licensing, free-living food intake, fasting appetite, and fasting appetite-related hormones were assessed using two-way (condition × time) ANOVAs. Insulin sensitivity was calculated using the homeostatic model assessment (HOMA-IR) index (based on fasting blood glucose and insulin concentrations) [30]. The effect of the exercise training on the responses of energy intake, perceived appetite, and appetite-related hormones, to an acute bout of exercise, were measured using two-way repeated measures (condition × time) ANOVAs. 

## 3. Results

### 3.1. Exercise Training

Training attendance was similar between groups, t(34) = 0.000, *p* = 1.000, with 35 ± 2 sessions completed in SIT and 35 ± 1 sessions in MICT of a total possible 36 sessions. Mean HR, *F*(1,34) = 0.074, *p* = 0.787, η^2^_p_ = 0.002, RPE, *F*(1,34) = 0.0036, *p* = 0.850, η^2^_p_ = 0.001, power, *F*(1,34) = 0.102, *p* = 0.752, η^2^_p_ = 0.003, and mechanical work, *F*(1,34) = 0.001, *p* = 0.972, η^2^_p_ = 0.000, were not significantly different between groups (Table 1). Perceived autonomy-support, *F*(1,33) = 15.651, *p* < 0.001, η^2^_p_ = 0.322, and structure (competence-support), *F*(1,33) = 4.245, *p* = 0.047, η^2^_p_ = 0.114, were higher in the SIT group compared with MICT, whereas involvement (relatedness-support) was not significantly different between groups, *F*(1,33) = 0.620, *p* = 0.437, η^2^_p_ = 0.018 (Table 1). Perceived enjoyment, *F*(1,34) = 11.523, *p* = 0.002, η^2^_p_ = 0.253, value, *F*(1,34) = 8.143, *p* = 0.007, η^2^_p_ = 0.193, and competence, *F*(1,34) = 14.698, *p* = 0.001, η^2^_p_ = 0.302, were higher in the SIT group, whereas choice, *F*(1,34) = 0.007, *p* = 0.932, η^2^_p_ = 0.000, and relatedness, *F*(1,34) = 3.726, *p* = 0.062, η^2^_p_ = 0.099, did not significantly differ between groups (Table 1).

### 3.2. Fitness, Body Composition, and Behavioral Characteristics

Participants’ physical characteristics were well-matched at baseline between groups, with no significant differences in age, *F*(1,34) = 1.130, *p* = 0.295, η^2^_p_ = 0.032, height, *F*(1,34) = 0.085, *p* = 0.733, η^2^_p_ = 0.002, body mass, *F*(1,34) = 0.004, *p* = 0.950, η^2^_p_ = 0.000, lean mass, *F*(1,34) = 0.035, *p* = 0.854, η^2^_p_ = 0.001, fat mass, *F*(1,34) = 0.246, *p* = 0.623, η^2^_p_ = 0.007, $\dot{V}O_{2\mathrm{peak}}$, *F*(1,34) = 0.328, *p* = 0.570, η^2^_p_ = 0.010, or baseline physical activity levels, *F*(1,34) = 0.487, *p* = 0.490, η^2^_p_ = 0.014 (Table 2).

Body mass, *F*(1,34) = 8.644, *p* = 0.006, fat mass, *F*(1,34) = 4.476, *p* = 0.042, η^2^_p_ = 0.116, and lean mass, *F*(1,34) = 10.257, *p* = 0.003, η^2^_p_ = 0.232, significantly increased from pre- to post-intervention; however, there were no differences between groups with no significant condition-by-interactions observed (*p* > 0.05). A significant main effect of time revealed an increase in $\dot{V}O_{2\mathrm{peak}}$, *F*(1,30) = 5.463, *p* = 0.026, η^2^_p_ = 0.154; however, again, the condition-by-time effect was non-significant, *F*(1,30) = 0.129, *p* = 0.722, η^2^_p_ = 0.004. Mean energy expended through physical activity outside of the exercise training, as measured by accelerometry over four days, did not differ pre- and post-intervention, *F*(1,24) = 1.570, *p* = 0.222, η^2^_p_ = 0.061, or between groups (no significant exercise training-by-time interaction), *F*(1,24) = 0.668, *p* = 0.422, η^2^_p_ = 0.027.

Participants’ self-reported motivation for exercise pre- and post-training is shown in Table 2. There was no effect of the time or condition-by-time interaction for amotivation, external regulation, introjected regulation, and integrated regulation (all *p* > 0.05). However, there was a significant effect of time on identified regulation, *F*(1,33) = 7.229, *p* = 0.011, η^2^_p_ = 0.180, with an increase from pre- to post-training, but no difference between groups (non-significant condition-by-time interaction), *F*(1,33) = 0.205, *p* = 0.654, η^2^_p_ = 0.006. Intrinsic motivation also significantly increased from pre- to post-training, *F*(1,33) = 7.680, *p* = 0.009, η^2^_p_ = 0.189, with the exercise condition-by-time interaction revealing greater increases in intrinsic motivation scores following SIT compared with MICT, *F*(1,33) = 6.791, *p* = 0.014, η^2^_p_ = 0.171.

### 3.3. Eating Behaviors, Fasting Appetite, and Fasting Appetite-Related Hormones

Self-reported compensatory eating and dietary restraint did not differ following the exercise training or between groups (all *p* > 0.05; Table 3). There was a trend for lower licensing post-training, *F*(1,33) = 3.648, *p* = 0.065, η^2^_p_ = 0.100, d = 0.43; however, the exercise condition-by-time effect was non-significant, *F*(1,33) = 0.000, *p* = 0.984, η^2^_p_ = 0.000. Mean daily free-living energy intake did not significantly differ following the exercise training, *F*(1,22) = 0.121, *p* = 0.731, η^2^_p_ = 0.005, or between groups, *F*(1,22) = 1.000, *p* = 0.328, η^2^_p_ = 0.043.

Appetite-related blood variables are shown in Table 4. There was no main effect of time, or condition-by-time interaction for fasting concentrations of glucose, lactate, ghrelin, leptin, insulin, or insulin sensitivity (all *p* > 0.05). Likewise, there was no effect of time or condition-by-time interaction for fasting hunger or fullness (all *p* > 0.05).

### 3.4. Appetite, Energy Intake, and Appetite-Related Hormones in Response to an Acute Bout of Exercise

For self-reported hunger there was a significant main effect of the acute exercise, *F*(1,33) = 23.272, *p* < 0.001, η^2^_p_ = 0.414, and a significant interaction between the acute exercise and condition, *F*(1,33) = 9.275, *p* < 0.001, η^2^_p_ = 0.414, such that hunger increased to a greater extent following SIT, compared with MICT, irrespective of the training. There were no other significant main or interaction effects for hunger, and there were no significant main or interaction effects for perceived fullness (all *p* > 0.05).

Energy intake on the day before Visit 2 pre- and post-training was well matched, such that no significant effects of condition, time, or condition-by-time were observed (all *p* > 0.05). Energy intake from the laboratory test meal pre- and post-training is shown in Table 5. There were no significant main effects of condition or time or condition-by-time interaction on total, ‘healthy’, or ‘unhealthy’ energy intake at the initial test meal (all *p* > 0.05).

Analysis of the total energy intake from snacks (i.e., for the remaining 2.5 h after the initial laboratory test meal) revealed no significant main effects of condition or time (*p >* 0.05); however, the condition-by-time interaction revealed a significantly higher total intake at baseline in SIT which decreased following the training, *F*(1,34) = 5.988, *p* = 0.020, η^2^_p_ = 0.150. There was no significant effects of condition, time, or condition-by-time on ‘healthy’ energy intake (all *p* > 0.05); however, there was a significant condition-by-time interaction effect on ‘unhealthy’ intake, *F*(1,34) = 10.314, *p* = 0.003, η^2^_p_ = 0.233, indicating a higher intake at baseline in SIT which decreased following the training (Table 5). 

## 4. Discussion

The primary aim of this study was to compare the effects of 12 weeks of SIT with psychological need-support and MICT without need-support on post-exercise energy intake. In immediate response to an acute bout of exercise (i.e., first 30 min), we observed that energy intake was not altered by 12 weeks of either SIT or MICT; however, total and ‘unhealthy’ energy intake from snacks in the subsequent 2.5 h following exercise was lower after 12 weeks of SIT, while unchanged in MICT. This attenuated food intake was associated with greater perceived enjoyment, value, and perceived competence of the SIT compared with the MICT intervention. With respect to the secondary objectives of this study, we observed no significant differences in fasting appetite or concentrations of appetite-related blood variables between groups.

This is the first investigation to consider the effects of exercise format, coupled with manipulations to the psychological conditions in which exercise is performed, on appetite responses following a multi-week exercise training intervention. Although energy intake at the immediate post-exercise (first 30 min) meal was not altered because of the exercise training, our finding that total—and specifically ‘unhealthy’—energy intake from snacks was lower following SIT, but not MICT. While total and ‘unhealthy’ snack intake was higher following the acute bout of SIT compared with MICT at baseline, more important to note is that the compensatory eating response to exercise decreased following 12 weeks of SIT, whereas post-exercise food consumption remained unchanged following 12 weeks of MICT. This attenuation in post-exercise food intake following SIT indicates a more appropriate energy compensation following exercise and is consistent with previous work in which a tendency for improved appetite regulation was observed following 12 weeks of SIT in physically inactive men with overweight [7]. Contrary to our findings, however, Miguet et al. [31] reported that the 24-h energy intake, assessed via buffet-style laboratory test meals, increased in a similar manner following 16 weeks of HIIT and MICT in adolescents with obesity, with no differences observed between groups. The differences in findings in the study by Miguet and colleagues [31] and our work, together with the data from Sim, Wallman, Fairchild and Guelfi [7], may be attributed to the population studied (i.e., predominantly women in our study as opposed to the men in the study by Sim et al. and adolescents in the study by Miguet et al. or the specific training protocol employed. Specifically, we utilized the same SIT protocol as Sim et al. which consisted of three cycling sessions per week, whereas the HIIT protocol in the study by Miguet, Fearnbach, Metz, Khammassi, Julian, Cardenoux, Pereira, Boirie, Duclos and Thivel [31] comprised of alternating cycling at 30 s of cycling at 75–90% $\dot{V}O_{2\mathrm{peak}}$ and free pedaling followed by strength training twice per week. It is possible that the greater energy expenditure associated with a longer duration (i.e., 30–50 min) of aerobic exercise in our study relative to the 15 min of HIIT completed in the study by Miguet, Fearnbach, Metz, Khammassi, Julian, Cardenoux, Pereira, Boirie, Duclos and Thivel [31] may exert a greater anorexigenic (i.e., suppressive) effect on appetite and subsequent energy intake due to the hormonal milieu associated with aerobic relative to resistance exercise [32] and/or adults and adolescents have different physiological responses to these forms of training. Importantly, our findings highlight the need for extended monitoring of food consumption (i.e., hours and days) as differences may only be evident beyond the immediate post-exercise meal.

With respect to perceived appetite and appetite-related blood variables, we observed no significant changes in fasting (pre-exercise) hunger or fullness in either group as a result of the training. This was not surprising given the lack of any significant changes in fasting active ghrelin, leptin, and insulin pre- to post-intervention. These findings are in line with those by Taylor et al. [33], who observed no significant changes in fasting concentrations of ghrelin and leptin following 4 weeks of HIIT (4 × 4 min exercise performed at 85–95% peak HR) or MICT (34 min of exercise performed at 65–75% peak HR) in cardiac rehabilitation patients. However, the absence of any changes to these appetite-related hormones contradicts previous work in which lower fasting insulin, insulin sensitivity, and leptin were observed in physically inactive men with overweight who completed the same 12-week SIT protocol as we employed in our study (albeit without the addition of psychological need-support) [7]. The discrepancies in leptin responses in our study and the study by Sim and colleagues may be explained by the notable (although not statistically significant) fat loss observed following SIT in the aforementioned study, given that exercise-induced weight loss is associated with attenuated leptin secretion [34] whereas participants in the current study gained fat mass in both exercise conditions, albeit a small amount. This may also explain the lack of improvement in fasting insulin and insulin sensitivity that we observed, despite the lower concentrations of fasting blood glucose observed following SIT (significant main effect of training). Regardless, our sample was comprised of predominantly women, whereas the study by Sim, Wallman, Fairchild and Guelfi [7] only included men, which may have also contributed to these discrepancies. With respect to the acute bout of exercise performed pre- and post-training, we noted a significant increase in perceived hunger following the acute bout of SIT and MICT, with this increase being greater in SIT. This was associated with significant decreases in circulating ghrelin and leptin following the acute exercise, independent of condition or whether the exercise was performed pre- or post-training (i.e., the response was not altered by training) and highlights the potential disconnect between appetite perceptions and appetite-regulating hormones. The greater increase in hunger from pre- to post-acute SIT, however, is consistent with our previous findings, whereby hunger increased following an acute bout of SIT and MICT [4]. However, the lower concentrations of leptin following the acute bout of SIT were not expected, given that leptin concentrations are not typically altered by an acute bout of exercise [35].

Interestingly, an increase in total, lean, and fat mass was observed in both groups as a result of the exercise training. While the mechanism for this is not initially clear, it is possible that the volume of exercise (i.e., up to 150 min per week) and associated energy expenditure may not have been enough to stimulate weight loss relative to potential compensatory responses which may have occurred, particularly given that this volume of exercise does not meet the ACSM guidelines for weight loss [36]. In a recent investigation comparing the effects of weekly exercise dose on energy compensation, Flack and colleagues [37] observed a similar increase in energy intake following their two 12-week exercise interventions expending 2754 kcal and 1491 kcal per week. Therefore, it is possible that the energy expenditure of the exercise training performed in our study was not great enough to counteract the potential upregulation of appetite, and in turn, stimulate weight loss. It is important to note, however, that self-reported compensatory eating was not altered as a result of the exercise training, indicating that any potential change to eating behaviors may have been non-conscious. Nonetheless, we observed an increase in aerobic fitness in our participants as a result of both SIT and MICT, which has important benefits for health, irrespective of weight loss [38].

Examination of participants’ psychological perceptions of the exercise training revealed that perceived autonomy and structure (competence) support were higher in SIT compared with MICT, as were perceptions of enjoyment, value, and competence. In line with these perceptions, we observed the formation of the most autonomous form of motivation among these participants with an increase in intrinsic motivation (i.e., enjoyment of exercise) following both interventions, with a greater increase observed in the SIT group. We also noted an increase in identified regulation (i.e., valuing the benefits of exercise) following both training interventions indicating that participation in these exercise programs, regardless of the format or psychological conditions, may have facilitated a more desirable profile of motivation toward exercise. The lack of significant difference in perceived involvement (relatedness-support) or relatedness satisfaction was surprising; however, this is in line with recent meta-analytic evidence, which has shown that interventions which aimed to shape psychological need-support were successful in increasing perceptions of overall need satisfaction, but not individually for relatedness satisfaction [39]. Regardless, the experiences and formation of more autonomous motivation may contribute to the decrease in total and particularly ‘unhealthy’ post-exercise snack consumption observed after the SIT intervention given our previous observations of attenuated total and unhealthy intake following an acute bout of exercise provided under autonomy-supportive conditions [19]. Although this study is the first to directly assess the influence of psychological need-support in a multi-week exercise intervention on appetitive responses, researchers have proposed possible ‘spill over’ effects in self-regulation, whereby increased autonomous exercise motivation may lead to improvements in eating self-regulation during weight control in women [17]. In their study, Mata and colleagues [17] invited women with overweight and obesity to participate in a 12-month exercise intervention that focused on promoting physical activity and internal (more autonomous) motivation for weight loss. These authors reported that the relationship between self-reported physical activity and healthy eating regulation was mediated by general self-determination, autonomous treatment motivation, and intrinsic exercise motivation. Although we did not observe a significant difference in self-reported compensatory eating following the exercise training, we did note a trend for lower exercise snacking licensing post-training across the two groups. The consistency between groups was surprising given that previous work has shown that individuals driven by more autonomous (relative to controlled) motivation may experience lower compensatory licensing beliefs [16]. How these results related to the reduced snack intake following SIT is unclear at this stage.

A strength of this study is the focus on men and women with overweight and obesity, as the effect of exercise training on appetite regulation may be of most relevance to this population. We purposefully recruited both men and women, given that prior research investigating the effect of exercise interventions has predominantly focused on men. However, we did not statistically power the study to investigate sex-specific responses to the exercise as this was not a primary aim of this study. Importantly, the yoked study design allowed us to standardize the characteristics of participants in the two groups at baseline, together with the power, mechanical work performed, and duration of exercise sessions such that all participants were completing 150 min of exercise per week in accordance with international minimum exercise guidelines towards the end of the intervention period. While the exercise conditions in this study were chosen due to the differences in energy intake seen following an acute bout of exercise [4], it is important to note that the effects of format and need-support cannot be isolated. That is, we cannot deduce whether the attenuated ‘unhealthy’ consumption of snacks following SIT was a direct result of the psychological need-support provided during exercise or the specific format of the exercise itself. Nonetheless, this study highlights the importance of considering the interaction of the psychological experiences and physiological demands of exercise, particularly given the concern over the prescription of HIIT protocols in sedentary populations [40]. The greater enjoyment reported by participants who completed SIT compared with MICT, together with the high attendance rate and similar RPE of the two exercise interventions, suggests that HIIT or SIT protocols, delivered in psychologically-need supportive conditions, may provide a suitable alternative for traditionally recommended moderate-intensity exercise in this population.

In summary, we have shown that 12 weeks of SIT with need-support resulted in significantly lower energy intake from ‘unhealthy’ snacks (compared with baseline) following an acute bout of exercise. The mechanisms behind this effect are unclear but may be associated with the increased perceptions of enjoyment, perceived value, and competence connected with this form of exercise. Together with previous evidence suggesting that an acute bout of SIT is well-tolerated and enjoyed by physically inactive individuals [4], findings from this work have important implications for current exercise prescription guidelines. Such considerations are particularly relevant to the format and psychological conditions of exercise, particularly in individuals where dietary intake is of concern.

## Figures and Tables

**Table 1:** Training session characteristics (physiological responses and psychological perceptions) of the 12-week SIT and MICT interventions (mean ± SD).

	MICT (*n* = 18)	SIT (*n* = 18)
Mechanical work (kJ)	244 ± 108	243 ± 92
Mean power (W)	88 ± 24	85 ± 24
Heart rate (bpm)	125 ± 9	124 ± 10
RPE	12 ± 2	12 ± 1
Need-support		
Autonomy	2.6 ± 0.9	3.6 ± 0.6 ^†^
Structure (competence)	3.3 ± 0.7	3.7 ± 0.5 ^†^
Involvement (relatedness)	3.6 ± 0.4	3.7 ± 0.4
Need-satisfaction		
Enjoyment	4.5 ± 1.5	5.9 ± 1.0 ^†^
Value	5.8 ± 1.2	6.7 ± 0.4 ^†^
Competence	4.9 ± 1.1	6.1 ± 0.8 ^†^
Choice	6.0 ± 1.0	6.0 ± 0.7
Relatedness	5.6 ± 0.8	6.3 ± 0.8

Note. MICT, moderate-intensity continuous training without need-support; SIT, Sprint interval training with need-support; RPE, Rating of Perceived Exertion. ^†^ Significant difference from MICT (*p* < 0.05).

**Table 2:** Descriptive characteristics of participants pre- and post- 12 weeks of SIT or MICT (mean ± SD).

Characteristic	Time Point	MICT (*n* = 18)	SIT (*n* = 18)
Age (years)	Pre	26.7 ± 7.0	29.2 ± 7.1
Height (cm)	Pre	168.8 ± 8.3	167.9 ± 10.0
Body mass (kg)	Pre	84.47 ± 15.14	84.16 ± 14.69
	Post ^†^	86.00 ± 15.93	84.88 ± 15.35
Fat mass (kg)	Pre	34.06 ± 9.26	35.53 ± 8.52
	Post ^†^	35.22 ± 10.07	35.65 ± 8.37
Lean mass (kg)	Pre	45.82 ± 9.97	45.21 ± 9.56
	Post ^†^	46.86 ± 9.86	46.45 ± 10.44
$\dot{V}O_{2\mathrm{peak}}$ (mL·kg·min^−1^)	Pre	21.18 ± 4.75	20.58 ± 3.56
	Post ^†^	24.47 ± 6.25	23.00 ± 3.86
Mean daily physical activity expenditure (kJ)	Pre	1569 ± 759	1697 ± 912
	Post	1196 ± 577	1619 ± 671
Motivation for exercise			
Amotivation	Pre	0.48 ± 0.65	0.44 ± 0.55
	Post	0.31 ± 0.56	0.25 ± 0.33
External regulation	Pre	1.25 ± 0.83	1.25 ± 0.99
	Post	1.25 ± 0.92	1.18 ± 1.10
Introjected regulation	Pre	2.56 ± 0.71	2.74 ± 0.89
	Post	2.29 ± 0.96	2.31 ± 1.09
Identified regulation	Pre	2.50 ± 0.51	2.61 ± 0.61
	Post ^†^	2.61 ± 0.72	2.98 ± 0.62
Integrated regulation	Pre	1.33 ± 0.64	1.57 ± 0.70
	Post	1.41 ± 0.88	1.92 ± 0.89
Intrinsic motivation	Pre	2.13 ± 0.81	2.10 ± 0.91
	Post ^†^	2.15 ± 0.96	2.74 ± 1.07 ^‡^

Note. MICT, moderate-intensity continuous training without need-support; SIT, Sprint interval training with need-support; BMI, body mass index. ^†^ Significant difference from pre-training (*p* < 0.05). ^‡^ Significant difference from MICT (*p* < 0.05).

**Table 3:** Self-reported eating behaviors before and after 12 weeks of SIT or MICT (mean ± SD).

	Time Point	MICT (*n* = 18)	SIT (*n* = 18)
Dietary restraint	Pre	2.84 ± 0.58	3.06 ± 0.81
	Post	2.79 ± 0.58	2.83 ± 0.75
CEMQ	Pre	2.21 ± 0.31	2.39 ± 0.42
	Post	2.29 ± 0.31	2.37 ± 0.50
ESLS	Pre	2.65 ± 0.90	2.79 ± 1.20
	Post	2.34 ± 0.90	2.47 ± 1.19
Mean daily energy intake (kJ)	Pre	6727 ± 1669	6967 ± 1362
	Post	7167 ± 3724	6059 ± 1410

Note. MICT, moderate-intensity continuous training without need-support; SIT, Sprint interval training with need-support; CEMQ, Compensatory Eating Motives Questionnaire; ESLS, Exercise Snacking Licensing Scale. No significant differences were observed between variables.

**Table 4:** Capillary concentrations of appetite-related blood variables in the fasting state (pre) and in response to 30 min of SIT and MICT exercise (post) performed before and after 12 weeks of SIT or MICT [(mean ± SD) or median (IQR)].

	Time Point (Pre- vs. Post-Exercise)	MICT (*n* = 18)	SIT (*n* = 18)
Pre-intervention			
Hunger (mm)	Pre	36 ± 22	36 ± 19
	Post	47 ± 22 ^‡^	58 ± 16 ^‡,^^§^
Fullness (mm)	Pre	29 ± 20	27 ± 21
	Post	25 ± 18	35 ± 23
Blood lactate (mM)	Pre	1.4 ± 0.6	1.2 ± 0.3
	Post	2.0 ± 1.1 ^‡^	3.4 ± 1.9 ^‡,^^§^
Blood glucose (mM)	Pre	4.0 ± 0.5	4.4 ± 0.6
	Post	3.9 ± 0.5	4.2 ± 0.7
Active ghrelin(pg·mL^−1^)	Pre	162 (112–206)	82 (28–116)
	Post	131 (71–183) ^‡^	44 (17–62) ^‡^
Insulin (pg·mL^−1^)	Pre	732 (549–956)	717 (567–947)
	Post	623 (537–924)	887 (835–1630)
Leptin (pg·mL^−1^)	Pre	11,326 (5108–17,154)	12,513 (8734–14,100)
	Post	8409 (4730–13,826) ^‡^	10,282 (7761–15,223) ^‡^
Post-intervention			
Hunger (mm)	Pre	50 ± 22	34 ± 29
	Post	49 ± 22 ^‡^	55 ± 21 ^‡,^^§^
Fullness (mm)	Pre	29 ± 22	30 ± 25
	Post	26 + 20	25 ± 20
Blood lactate (mM)	Pre	1.4 ± 0.6	1.2 ± 0.2
	Post	2.2 ± 1.1 ^‡^	2.6 ± 1.4 ^‡,^^§^
Blood glucose (mM)	Pre	3.9 ± 0.6 ^†^	4.1 ± 0.8 ^†^
	Post	3.8 ± 0.7 ^†^	3.7 ± 0.4 ^†^
Active ghrelin (pg·mL^−1^)	Pre	147 (79–203)	66 (39–98)
	Post	138 (91–186) ^‡^	49 (23–70) ^‡^
Insulin (pg·mL^−1^)	Pre	615 (453–888)	894 (453–1377)
	Post	649 (510–943)	903 (755–1430)
Leptin(pg·mL^−1^)	Pre	11,274 (7741–14,332)	10,866 (8310–13,841)
	Post	9458 (5675–14,491) ^‡^	9493 (7490–11,303) ^‡^

Note. MICT, moderate-intensity continuous training without need-support; SIT, Sprint interval training with need-support; ^†^ Significant difference from pre-intervention (*p* < 0.05). ^‡^ Significant difference from pre-exercise (*p* < 0.05). ^§^ Significant difference from MICT (*p* < 0.05).

**Table 5:** Energy intake from a laboratory test meal (initial 30 min) and from snacks (following 2.5 h) following 30 min of SIT or MICT performed pre- and post- 12 weeks of training.

Variable	Time Point (Pre- vs. Post-Training)	MICT (*n* = 18)	SIT (*n* = 18)
Total energy intake from initial test meal (kJ)	Pre	1742 ± 997	1922 ± 775
	Post	1944 ± 1481	2074 ± 854
‘Healthy’ energy intake from initial test meal (kJ)	Pre	1127 ± 745	979 ± 629
	Post	1185 ± 841	1065 ± 585
‘Unhealthy’ energy intake from initial test meal (kJ)	Pre	615 ± 776	928 ± 660
	Post	753 ± 885	990 ± 766
Total energy intake from snacks (kJ)	Pre	376 ± 565	807 ± 550 ^‡^
	Post	444 ± 824	422 ± 468 ^†^
‘Healthy’ energy intake from snacks (kJ)	Pre	110 ± 183	77 ± 177
	Post	61 ± 141	116 ± 181
‘Unhealthy’ energy intake from snacks (kJ)	Pre	261 ± 582	730 ± 533 ^‡^
	Post	383 ± 841	307 ± 393 ^†^

Note. MICT, moderate-intensity continuous training without need-support; SIT, Sprint interval training with need-support; ^†^ Significant difference from pre-training (*p* < 0.05). ^‡^ Significant difference from MICT (*p* < 0.05).

## Data Availability

Not applicable.
